# Malaria Parasitemia in Febrile Patients Mono- and Coinfected with Soil-Transmitted Helminthiasis Attending Sanja Hospital, Northwest Ethiopia

**DOI:** 10.1155/2020/9891870

**Published:** 2020-02-04

**Authors:** Fantahun Getaneh, Ayalew Jejaw Zeleke, Wossenseged Lemma, Yalewayker Tegegne

**Affiliations:** ^1^Shegaw Mota Hospital, Ethiopia; ^2^Department of Medical Parasitology, School of Biomedical and Laboratory Sciences, College of Medicine and Health Sciences, University of Gondar, Gondar, Ethiopia

## Abstract

**Background:**

Malaria is a life-threatening disease associated with high morbidity and mortality. Helminths are among the most widespread infectious agents prevalent in tropical and subtropical regions of the developing world. Malaria and soil-transmitted helminthiasis (STHs) are coendemic and major public health problems in Ethiopia. The effects of helminth coinfection on malaria parasitemia remained poorly understood. Therefore, the objective of this study was to assess malaria parasitemia among malaria-monoinfected and malaria-soil-transmitted helminthiasis–coinfected febrile patients attending Sanja Hospital, Northwest Ethiopia.

**Methods:**

A cross-sectional study with parallel groups was conducted to assess malaria parasitemia among malaria-monoinfected and malaria-soil-transmitted helminthiasis–coinfected febrile patients in Sanja Hospital from January to March 2019. Double population proportion formula was used for sample size calculation, and convenient sampling technique was used to select 134 study participants. Data were entered and analyzed by using the Statistical Package for Social Sciences (SPSS) version 20. Descriptive statistics, independent *t*-test, and one-way analysis of variance (ANOVA) were performed. A *P* value of <0.05 was considered as statistically significant.

**Results:**

From 134 malaria-positive study participants, 67 were malaria-monoinfected and 67 were malaria-STHs–coinfected patients. Out of 67 malaria STHs-coinfected patients, 54 (80.6%) were infected with hookworm followed by *Ascaris lumbricoides* 11 (16.4%) and *Strongyloides stercoralis* 2 (3%). The mean *Plasmodium* parasite density was significantly higher in malaria-STHs–coinfected patients than in patients infected with only *Plasmodium* parasite density was significantly higher in malaria-STHs–coinfected patients than in patients infected with only *P* value of <0.05 was considered as statistically significant. *Plasmodium* parasite density was significantly higher in malaria-STHs–coinfected patients than in patients infected with only *F* = 6.953, *P* value of <0.05 was considered as statistically significant.

**Conclusions:**

Infections with STHs, especially hookworm, were positively associated with *Plasmodium* parasite density. The current study finding also revealed that increased worm burden of hookworm as expressed by egg intensity had significantly increased *Plasmodium* parasite density.*Plasmodium* parasite density was significantly higher in malaria-STHs–coinfected patients than in patients infected with only *Plasmodium* parasite density was significantly higher in malaria-STHs–coinfected patients than in patients infected with only

## 1. Introduction

Malaria is a disease caused by a protozoan parasite belonging to the genus *Plasmodium* and transmitted by different species of female *Anopheles* mosquitoes. The five known species of *Plasmodium* parasites that cause malaria for humans are *Plasmodium falciparum* (*P. falciparum*), *Plasmodium vivax* (*P. vivax*), *Plasmodium ovale* (*P. ovale*), *Plasmodium malariae* (*P. malariae*), and *Plasmodium knowlesi* (*P. knowlesi*) [1]. The *Plasmodium* parasites which cause malaria involve two hosts in their life cycle. During blood feeding, a malaria-infected female *Anopheles* mosquito inoculates sporozoites into the human host [2].

Human intestinal helminthiasis is most commonly caused by soil-transmitted helminths (STHs), namely, *Ascaris lumbricoides* (*A. lumbricoides*), *Trichuris trichiura* (*T. trichiura*), and the hookworms *Necator americanus* and *Ancylostoma duodenale* [3]. Soil-transmitted helminth infections are among the most common infections worldwide and widely distributed in tropical and subtropical areas with the greatest numbers found in Sub-Saharan Africa (SSA), East Asia, South America, China, and India [4]. Transmission occurs through eggs and larvae developing in contaminated soil with feces containing helminth eggs [5].

Malaria and helminthiases are the two most common predominant infections affecting humans, overlapping in their epidemiological distributions and frequently coinfecting the same individuals [6]. The occurrence of their coinfection results from similar environmental covers of coinfecting species that increase exposure-related risks of coinfection [7].

Different immunological mechanisms induced by helminth infection have been emphasized as potentially protective against *Plasmodium* infection or increasing the risk. Helminths are believed to have greater generalized immunoregulatory consequences than their copathogens such as *Plasmodium*. During their lifetime, helminths do not simply ward off the immune attack but they regulate the host immune response to create niches that improve successful feeding and reproduction [8].

Chronic helminth infection may enforce crossregulatory effects on the development of proinflammatory response to initial *Plasmodium* infection and skewed antiplasmodium antibody response towards the production of noncytophilic immunoglobulins (IgG2, IgG4, and IgM) ineffective against malaria instead of cytophilic ones necessary for immunity of malaria (IgG1 and IgG3). This will lead to an increased incidence and severity of malaria. Another explanation for the observed interaction of malaria and STHs is that T cell with the regulatory function may be induced in helminthiasis-infected patients leading to suppression of Th1 cells and proinflammatory activity [9].

There are a number of studies that assessed the magnitude of coinfection between malaria and STHs; however, there are no adequate reports which show the association of coinfection with the level of malaria parasitemia. Even though both malaria and STHs are common in Sanja town and the surrounding area due to the low land geographical nature of the area, there have been no studies conducted similarly to this study. Therefore, the present study was conducted to assess the association of STHs infection with malaria parasitemia in febrile patients attending Sanja Hospital, Northwest Ethiopia.

## 2. Methods

### 2.1. Study Area, Design, and Period

An institutional-based comparative cross-sectional study was conducted to assess malaria parasitemia level among malaria-monoinfected and malaria-soil-transmitted helminthiasis–coinfected febrile patients attending Sanja Hospital, Northwest Ethiopia. Sanja is the capital of Tach Armachiho district which is surrounded by the Maho Stream and Sanja River. The town is located 65 km in the North of Gondar town and 792 km from Addis Ababa, northwest part of Ethiopia. Sanja has an altitude of 1800 m above sea level, with an annual rainfall range from 800 to 1800 mm, and the annual temperature range from 25°C to 42°C [10]. An estimated rural and urban total population of Sanja woreda is 159,696, and Sanja town has a population of 3591 males and 3664 females which total into 7255 inhabitants [11]. One health center and one hospital give a service for the dwellers of the town and surrounding areas. The study was carried out from January to March 2019.

### 2.2. Sample Size Determination and Sampling Technique

The required sample size (*n*) was calculated by using a double population proportion formula with the assumptions of 95% confidence level, 5% of marginal error, 80% power (1 − *β*), and using prevalence of malaria, *p*_1_ = 16% (from the study conducted in Dilla, Ethiopia) [12], and prevalence of malaria-STHs coinfection, *p*_2_ = 5% (from the study conducted in Azezo, Ethiopia) [13].

(1) $$\begin{array}{c} n=\frac{2\times \left(Р\right)\left(1-P\right)\times \left(\text{Z}\beta +\text{Z}\alpha /2\right)2}{\left(p_{1}–p_{2}\right)2}, \end{array}$$

where *n* is the required sample size.

Therefore, the minimum required sample size was 122. By adding 10% for nonrespondent, we had the final sample size = 134 (67 for each group). Convenient sampling technique was used to select the study participants until the expected sample size was obtained.

### 2.3. Data Collection and Laboratory Methods

#### 2.3.1. Sociodemographic Data

The sociodemographic characteristics were collected by trained data collectors and supervised by the principal investigator. All febrile patients suspected of having malaria in Sanja Hospital were the source population of this study. Before data collection, data collectors selected patients who fulfilled the inclusion criteria and taken consent or assent form. If the study participants were children, consent was taken from their parent or guardian. All laboratory confirmed malaria-positive patients who had the ability to give stool sample, and patients greater than 6 months of age were included in the study. Patients who had a history of antihelminthic treatment in the past four weeks prior to screening, had any parasitic infection other than STHs in addition to malaria, are pregnant or lactating mothers, and had the presence of any other known chronic infections were excluded from the study.

### 2.4. Sample Collection and Laboratory Procedure

#### 2.4.1. Blood Film Parasite Detection

Thick and thin smears were prepared on a single slide for each acute febrile patient from capillary blood by finger prick using sterile lancet. Each blood smear was air dried and stained with Giemsa and examined under the oil immersion microscope objective. A hundred fields were examined before reporting a negative result. Thick smear was used to detect *Plasmodium* infection and parasite quantification. The thin smear was used to identify the type of *Plasmodium* species. The number of parasite per microliter of blood was calculated as described in [14]. It was calculated by counting the number of asexual parasites in a set number of white blood cells (WBCs) (commonly 200) with a hand tally counter. Once a field has been started, it must be counted until a field has been completed. If more than 500 parasites have been counted before 200 WBCs have been reached, the count was stopped after reading the last field that has been completed, and if fewer than 10 parasites have been counted per 200 WBCs, the reading was continued up to 500 WBCs [14]. Parasite density expressed as the number of asexual parasites per *μ*l of blood was calculated by dividing the number of asexual parasites by the number of WBCs counted and then multiplying by an assumed WBCs per *μ*l of blood (standard number of WBC = 8000/*μ*l).

(2) $$\begin{array}{c} \frac{\text{Parasite density}}{\mu \text{l}}=\frac{\text{Number of parasites counted}}{\text{Number of leukocytes counted}}\times \left(8000\right). \end{array}$$

Two qualified laboratory technologists had read all the slides independently, and the parasite densities were calculated by averaging the two counts.

### 2.5. Collection of Stool Samples and Examination for STH Infection

Approximately 5 gm of fresh stool specimen was collected by using labeled screw-capped stool cup from each participant; then, wet mount and two Kato slides per sample were prepared for microscopic examination. Quantitative examination for STH was performed, and the average egg counts of the two Kato Katz slides were multiplied by 24 to obtain egg counts per gram of stools for each study participant [15]. Egg counts were utilized to classify infection intensities into light, moderate, or heavy infections, respectively: *A. lumbricoides*, 1–4999 EPG, 5000–49999 EPG, and ≥50000 EPG; *T. trichiura*, 1–999 EPG, 1000–9999 EPG, and ≥10000 EPG; and hookworm, 1-1999 EPG, 2000-3999 EPG, and ≥4000 EPG [16].

### 2.6. Quality Control

The questionnaire was pretested on patients from Tikl Dengay Health Center for its accuracy and consistency prior to the actual data collection, and appropriate one day training was given to data collectors about the objectivity and relevance of the study, confidentiality issues, study participants' rights, consenting, techniques of an interview, and laboratory test procedures and their quality control. Giemsa stain quality was checked by using the known positive and negative samples for every batch of prepared working solution. After the data collection process, the data were checked for completeness and any incomplete or misfiled questionnaires were checked again by the principal investigator. For quality purposes, 10% of Giemsa-stained and Kato Katz slides were reexamined by an experienced laboratory technologist who was blind for the first result [17, 18].

### 2.7. Data Management and Analysis

The data were checked before entering for analysis for its completeness. Then, data were entered into SPSS version 20.0 for analysis purposes. Before analysis, data were cleaned or checked for errors and missing observations. Descriptive statistics was used to give a clear picture of background variables like age and sex. The frequency distributions of independent variables were done. An independent *t*-test was used to show the mean differences of *Plasmodium* parasite density between *Plasmodium*-STH coinfected and those who were infected with *Plasmodium* parasites alone. One-way ANOVA was used to test mean differences of parasitemia with intensity level of STHs infection. *P* values less than 0.05 were considered as statistically significant.

## 3. Results

### 3.1. Sociodemographic Characteristics of the Study Participants

A total of 2675 malaria-suspected patients visited Sanja Hospital from January to March 2019. From the total screened for malaria, 512 (19.1%) were found positive for *Plasmodium* parasites in which 242 (47.3%) were males and 270 (52.7%) were females. Among 512 malaria-positive patients who provided stool samples for STH detection, 134 study participants who fulfilled the inclusion criteria were included in the study. During blood smear examination, there were no discordant results, i.e., there were no differences between the two microscopists in species diagnosis, in parasite density of >50% discrepancy, or in the presence of parasites.

From the total of 134 *Plasmodium*-infected study participants, 72 (53.7%) were females. The mean (±SD) age of the study participants was 27 (±14) years and 25 (±14) years for malaria-monoinfected and malaria-STHs–coinfected patients, respectively. The majority, 92 (68.7%), of the study participants were from rural residences, 74 (55.2%) were farmers and 80 (59.7%) of them were unable to read and write. The predominant age group of the study participants was 15-45 years (73.1%) (Table 1).

### 3.2. Malaria Parasitemia Level among Malaria-Monoinfected and Malaria-STHs–Coinfected Study Participants

Among the total of 134 malaria-positive study participants, 67 were only *Plasmodium* species parasite-infected and the other 67 were malaria-STHs–coinfected patients. The majority of the infection was due to *P. falciparum* 85 (63.4%) followed by *P. vivax* which is accounted for 38 (28.4%) of the cases and the remaining 11 (8.2%) were mixed infection with *P. falciparum* and *P. vivax*. The mean malaria parasitemia level in malaria-monoinfected and malaria-STHs–coinfected patients was 7543.12 and 11909.80, respectively. There was statistically significant differences in mean parasite density of *Plasmodium* among malaria-monoinfected and malaria-STHs–coinfected study participants. The mean parasitemia level was significantly higher in malaria-STHs–coinfected patients than in patients infected with only *Plasmodium* parasites (*P* = 0.027) (Table 2).

### 3.3. Association between Malaria Parasitemia and Soil-Transmitted Helminthiasis

From the total of 67 malaria-STHs–coinfected study participants, the most frequent cause infection was hookworm which accounted for 54 (80.6%) followed by *A. lumbricoides* 11 (16.4%) and *S. stercoralis* 2 (3%). Moreover, from malaria-STHs–coinfected study participants, *Plasmodium* parasite density was high in patients coinfected with hookworm followed by those coinfected with *A. lumbricoides* and *S. stercoralis* (*F* = 0.356, *P* = 0.702), i.e., the mean *Plasmodium* density in coinfection with hookworm, *A. lumbricoides*, *and S. stercoralis* was 15063.64, 11330.67, and 10200.00, respectively. However, the difference was not statistically significant (Table 3).

### 3.4. Association between Malaria Parasitemia and Intensity of Soil-Transmitted Helminthiasis

Out of 54 malaria-hookworm–coinfected study participants, 66.7% (36/54), 18.5% (10/54), and 14.8% (8/54) had light, moderate, and heavy hookworm infection, respectively, and out of 11 participants infected with *Plasmodium and A. lumbricoides*, 54.5% (6/11), 27.3% (3/11), and 18.2% (2/11) had light, moderate, and heavy infection with *A. lumbricoides*, respectively. As the intensity of hookworm infection increased, mean *Plasmodium* density was also increased in coinfected patients. The difference was statistically significant (*F* = 6.953, *P* = 0.002). Additionally, the study participants who had heavy hookworm infection had significantly higher *Plasmodium* densities as compared to lightly or moderately infected patients. However, the mean levels of *Plasmodium* density decrease as intensity increased in *A. lumbricoides*-infected cases even though the difference was not statistically significant (*F* = 0.640, *P* = 0.552) (Table 4).

## 4. Discussion

Approximately 27% and 41% of the total population in Ethiopia live in high and low malaria transmission areas, respectively [19]. Moreover, most of the time, malaria and STHs infections share endemicity in Ethiopia [20–22]. Evidences suggested that the *Plasmodium* density had a heterogeneous association with different species of STHs in malaria-STHs–coinfected patients [23, 24].

The present study showed that the mean parasitemia density of *Plasmodium* was higher in malaria-STHs–coinfected patients as compared with patients infected with *Plasmodium* parasite alone. This indicates that helminth infection increases some degree of risks for the development of malaria severity by increasing parasite density of the patients since high parasite density could be a potential factor for the development of severe malaria. This result is also in agreement with the previous studies conducted in Southern Ethiopia [20] and findings of a meta-analysis [25]. Additionally, a systematic review and meta-analysis done in Sub-Saharan Africa showed that *P. falciparum* density inclined to be higher among children infected with STHs than those which are not infected with STHs. This might be due to the fact that helminths modulate the host immune response both to themselves and to coexisting infections or to the downregulation of the immune system by helminths. Consequently, the *Plasmodium* parasite could enter into the host and multiply at a faster rate in patients coinfected with STHs [26].

The present findings appear to verify the hypothesis that clinical malaria can be intensified during concomitant infection of intestinal helminths and *Plasmodium* parasites [27]. Helminths are known to induce regulatory T cells leading to the production of cytokines that counteract Th1 response and also modulate the function of dendritic cells. As a result, there is altering of the immune response to *Plasmodium* antigen [6]. Worm infections could also make the skin less retort to mosquito bites promoting the success of sporozoite to pass through it and increasing the chance of blood stage infection [28]. However, the result was in contrast with the previous studies conducted in Ethiopia and in different locality school children of Tanzania [29–31]. The observed differences may be due to the differences in the nature of the study participants (children, adults), immune status of the study participants, infecting *Plasmodium* species, and complex nature of helminth and *Plasmodium* interaction when they coexist in a host.

The current study also demonstrated that malaria with hookworm coinfection showed higher *Plasmodium* density level than coinfection with *A. lumbricoides*. This means that hookworm is associated with increased *Plasmodium* density which suggests a positive association between hookworm infection and occurrence of severe malaria. The finding is in agreement with the previous reports [26, 29]. In contrary, *A. lumbricoides* infection showed less *Plasmodium* density level than coinfection with hookworm which associated with reducing malaria severity. This finding was comparable with the studies which showed the protective effect of *A. lumbricoides* to reduce the severity of malaria [24, 32]. This paradoxical association can be explained by different biological hypotheses as follows.

The first hypothesis for the association of helminths with protection from severe malaria was that helminths led to an increase in IgE complexes that activated the CD23 and thus releases the anti-inflammatory IL10 and activated the inducible nitric oxide synthase, which led to the release of nitric oxide (NO) and reduced sequestration of parasitized red blood cells [33].

The second hypothesis is that worms decrease cytophilic IgG1 and IgG3 antibodies, which are necessary for malaria immunity, whereas it increased the noncytophilic IgG2, IgG4, and IgM antibodies. Consequently, it leads to an increase of severity of malaria [34].

In the current study, study participants infected lightly with hookworm had significantly lower *Plasmodium* densities compared to heavily infected patients. Moreover, the study participants who had heavy hookworm infection had significantly higher *Plasmodium* densities as compared to lightly or moderately infected patients. Thus, a positive association between worm burden as expressed by egg intensity and *Plasmodium* parasite density was observed, which suggests that with the time course of the infection, such patients with high density of both hookworm and *Plasmodium* will tend to develop severe form of clinical malaria by influencing the immune response during coinfection [35]. However, light infection with *A. lumbricoides* was associated with higher *Plasmodium* parasitemia, while those with heavy *A. lumbricoides* infection were found to have lower parasitemia, suggesting a positive interaction between hookworm infection intensity with *Plasmodium* densities whereas negative association between *A. lumbricoides* intensity with *Plasmodium* densities.

This finding was comparable with the previous studies that were conducted in different parts of Ethiopia like Alaba Kulito and Gilgel Gibe which suggested that STH infections especially hookworm aggravate the severity of malaria [21, 29]. However, the finding was contradicted with the study conducted in Nigeria and Wondo Genet, Ethiopia [22, 36]. These controversial results on mean *Plasmodium* density changes could have arisen due to the differences in the study groups, immune status of the study participants, differences in infecting *Plasmodium* species, differences in residence country, or complex nature of helminth and *Plasmodium* interaction when they coexist in a host.

## 5. Conclusions

In the current study, infections with STHs were positively associated with *Plasmodium* parasite density. The current study finding also revealed that increased worm burden of hookworm as expressed by egg intensity was associated with increased *Plasmodium* density which could be a density-dependent risk factor for the development of severe malaria with the course of the disease.

## Figures and Tables

**Table 1:** Sociodemographic characteristics of the study participants, Northwest Ethiopia, 2019.

Variables		Malaria-monoinfected patients *n* (%)	Malaria-STHs–coinfected patients *n* (%)	Total *n* (%)
Sex	Male	32 (47.8)	30 (44.8)	62 (46.3)
	Female	35 (52.2)	37 (55.2)	72 (53.7)

Age (years)	<15	9 (13.4)	12 (17.8)	21 (15.7)
	15-45	50 (74.6)	48 (71.6)	98 (73.1)
	>45	8 (11.9)	7 (10.5)	15 (11.2)

Residence	Urban	25 (37.3)	17 (25.4)	42 (31.3)
	Rural	42 (62.7)	50 (74.6)	92 (68.7)

Educational level	Unable to read and write	39 (58.3)	41 (61.2)	80 (59.7)
	Primary school	16 (23.9)	16 (23.9)	32 (23.9)
	High school and above	12 (17.8)	10 (14.9)	22 (16.4)

Occupation	Farmer	36 (53.7)	38 (56.7)	74 (55.2)
	Merchant	9 (13.4)	7 (10.4)	16 (11.9)
	Government employee	6 (9)	5 (7.5)	11 (8.2)
	Others^∗^	16 (23.9)	15 (25.4)	33 (24.6)

Note: STHs: soil-transmitted helminthiasis. ^∗^Includes student, daily laborer, and housewife.

**Table 2:** *Plasmodium* parasite density among malaria-monoinfected and malaria-soil-transmitted helminthiasis–coinfected study participants at Sanja Hospital, Northwest Ethiopia, from January to March 2019 (*N* = 134).

	Parasitemia of participant	*n*	Mean	Std. deviation	Std. error mean
Parasite density	Malaria-monoinfected	67	7543.12	8541.292	1043.485
	Malaria-STHs–coinfected	67	11909.79	13550.258	1655.427

Note: STHs: soil-transmitted helminthiasis; SD: standard deviation.

**Table 3:** Association between malaria parasitemia and soil-transmitted helminthiasis infection among malaria- and helminthiasis-coinfected patients at Sanja Hospital, Northwest Ethiopia, from January to March 2019 (*N* = 67).

Malaria-STHs coinfection	Mean (±SD) parasitemia	95% CI	ANOVA (one-way)
Malaria with hookworm (54)	15063.64 ± 14628.96	5235.76, 24891.51	*F* = 0.356
Malaria with *A. lumbricoides* (11)	11330.67 ± 13620.24	7613.06, 15048.28	*P* = 0.702
Malaria with *S. stercoralis* (2)	10200 ± 3563.81	-21819.64, 42219.64	

Note: STHs: soil-transmitted helminthiasis; SD: standard deviation; CI: confidence interval.

**Table 4:** Association between intensity of soil-transmitted helminthiasis infection and parasitemia among malaria- and helminthiasis-coinfected patients at Sanja Hospital, Northwest Ethiopia, from January to March 2019 (*N* = 67).

STHs		Mean parasitemia	95% CI	ANOVA (one-way)	Gabriel, multiple comparison
Hookworm	Light	8392.50^a,b^	4741.74, 12043.26	*F* = 6.953	*P* = 0.001^∗^, 0.96
	Moderate	10244.60^a^	1239.78, 19249.42	*P* = 0.002^∗^	
	Heavy	26230.00	11427.72, 41032.28		*P* = 0.024^∗^

*A. lumbricoides*	Light	19706.67	13399.25, 39274.08	*F* = 0.640	
	Moderate	12433.33	-178.80, 25044.57	*P* = 0.552	
	Heavy	6580.00	-52630.91, 65780.91		

Note: STHs: soil-transmitted helminthiasis; CI: confidence interval; ^∗^statistically significant. a = as compared to heavy hookworm infection; b = as compared to moderate hookworm infection.

## Data Availability

I confirmed that all the data for this manuscript are available, if someone wants to request the data can contact the Yalewayker Tegegne.

## Abbreviations

ANOVA: Analysis of variance
DALYs: Disability adjusted life years
EPG: Egg counts per gram
IFN: Interferon
IL: Interleukin
SPSS: Statistical Package for Social Sciences
SSA: Sub-Saharan Africa
STHs: Soil-transmitted helminths
TNF: Tumor necrosis factor
WBCs: White blood cells
WHO: World Health Organization.
